# Diagnostic Accuracy of Endoscopic Ultrasonography in Selecting Patients for Endoscopic Submucosal Dissection for Early Gastrointestinal Neoplasms

**DOI:** 10.3390/jcm12072505

**Published:** 2023-03-26

**Authors:** Pietro Gambitta, Paola Fontana, Ilaria Fanetti, Giulia Veglia, Maurizio Vertemati, Antonio Armellino, Paolo Aseni

**Affiliations:** 1 Department of Gastroenterology, Ospedale Civile di Legnano, ASST-Ovest Milanese, 20025 Legnano, Italy; 2 Department of Biomedical and Clinical Sciences “L. Sacco”, Università degli Studi di Milano, 20157 Milan, Italy; 3 Endoscopy Division, Ospedale San Leopoldo Mandic di Merate, ASST Lecco, 23807 Lecco, Italy; 4 Department of Emergency, ASST Grande Ospedale Metropolitano Niguarda, 20162 Milan, Italy

**Keywords:** subepithelial lesion, endoscopic ultrasonography, prognosis, gastrointestinal neoplasms, ESD, staging

## Abstract

Tumor invasion depth and lymph node metastasis determine the prognosis of gastrointestinal (GI) neoplasms. GI neoplasms limited to mucosa (m1 or m2) and superficial submucosa (sm1) can be treated effectively with minimally invasive endoscopic therapy, while the deep invasion of the submucosa (sm2 or sm3) is associated with lymph node metastasis, and surgical resection is required. Correct staging is therefore crucial for preoperative evaluation and planning. Endoscopic ultrasonography (EUS) can be used to detect the depth of invasion due to its close proximity to the lesion. The diagnostic accuracy of EUS, when compared to conventional endoscopic staging, is debated as it can under- or overstage the lesion. We aim in this study to determine if EUS can accurately differentiate mucosal from submucosal GI neoplasms to select patients with early GI lesions for endoscopic submucosal dissection (ESD) or surgery. From March 2014 to February 2022, 293 patients with early superficial GI neoplasms were admitted to our endoscopic unit for EUS staging. To evaluate the accuracy of EUS, we compared the preoperative EUS findings with the definitive histopathologic findings on the resected specimen. Overall, 242 of 293 lesions were correctly staged by EUS (82.59%). In the evaluation of submucosal invasion or deeper, EUS understaged 38 of 293 (12.96%) and overstaged 13 of 293 (4.43%) lesions. EUS has excellent accuracy in staging superficial GI neoplasms; its use is highly recommended before ESD since it can also detect lymph node metastases around the lesions, thus changing the indication from ESD to surgery.

## 1. Introduction

The widespread use of screening endoscopy and improvements in endoscopic techniques, such as narrow-band imaging, chromoendoscopy, and high-magnification endoscopy, have enabled the identification of superficial lesions such as faint mucosal irregularity or discoloration, previously overlooked; therefore, superficial GI neoplasms are more frequently diagnosed [1,2]. The prognosis of GI cancer depends on the depth of tumor invasion and lymph node metastasis (LNM). There is a strong relationship between the depth of tumor invasion and lymph node metastasis in superficial GI neoplasms [3,4,5].

The risk of regional LNM in the esophagus rises from less than 5% to about 20% with increasing depth of vertical invasion into the submucosal (sm) layer because the amount of lymphovascular supply increases deeper in the sm layer (sm2, sm3) [3,4].

Early GI cancers are defined as being limited to the mucosa or submucosa but not invading the *muscularis propria*, regardless of the presence of lymph node metastases. A macroscopic classification of these lesions was first established by Japanese endoscopists, and it was ratified at the Paris workshop in 2002 [5,6]. It has now been accepted worldwide and reported by the American Joint Committee on Cancer as the most accepted staging classification [7].

Superficial esophageal cancer (SEC) involving only the mucosa (T1a) or the superficial layer of the submucosa has less than a 5% to 9% chance of metastasis, compared with a 19% to 44% chance of lymph node metastasis with SEC invading the deep submucosa (T1b) [8,9,10,11]. Similarly, early gastric cancer (EGC) involving only the mucosa (T1m) or the superficial layer of the submucosa has a 0% to 2.6% chance of lymph node metastasis, compared with a 5% to 19% chance of lymph node metastasis with EGC invading the deep layer of the submucosa (sm2–sm3) [12,13]. In contrast, early cancer of the colon and rectum involving the mucosa or the superficial layer of the submucosa has a very low likelihood of metastasizing to lymph nodes and represents an indication for endoscopic resection [14,15].

Endoscopic submucosal dissection (ESD) is an innovative advance in the treatment of early GI cancer without lymph node metastases, but preoperatively correct staging is crucial [16]. A T1m or T1sm1 GI cancer can be effectively treated with endoscopic therapy, as opposed to T1sm2-sm3 cancers or deeper that are treated as advanced cancer [16,17].

Compared to conventional endoscopic staging, EUS can under- or overstage the lesion, and diagnostic accuracy is controversial. Recently, the accuracy of the EUS has been questioned by several authors [18,19]. The aim of the current study is to evaluate the accuracy of EUS in differentiating submucosal from superficial GI cancer.

## 2. Methods

Between March 2014 and February 2022, 293 patients who presented with a superficial epithelial lesion during a screening endoscopy were submitted to staging EUS to select the appropriate treatment (endoscopic resection vs. surgical resection).

The screening endoscopic examinations were considered positive if the biopsy results indicated low-grade dysplasia, high-grade dysplasia, or adenocarcinoma according to the Vienna classification of GI epithelial neoplasia [20]. Of 293 patients, 81 (27.64%) had noninvasive low-grade neoplasms, 90 (30.71%) had noninvasive high-grade neoplasms, 114 (38.90%) had invasive adenocarcinomas, and in 8 patients (2.73%) a carcinoid tumor was detected (Table 1).

**Figure 1:**
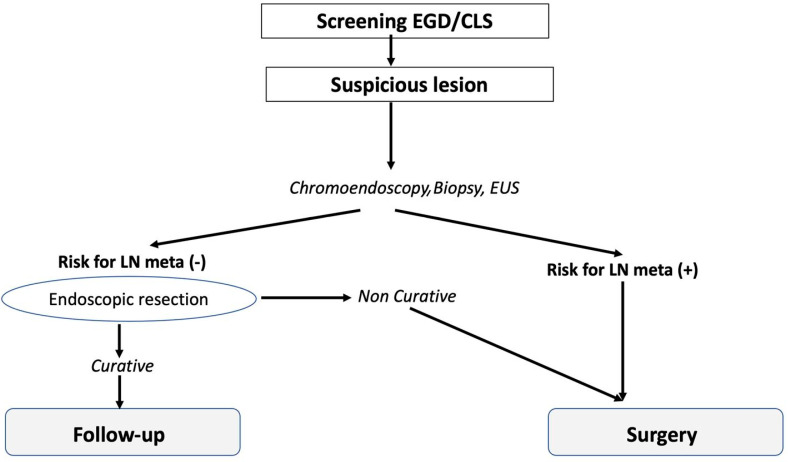
Diagnostic flowchart for patients with suspected superficial GI cancer to select those eligible for ESD or surgery. (LN—lymph node; CLS—colonoscopy).

In our endoscopic unit, a simple flowchart (Figure 1) was developed for all patients suspected of having GI cancer according to suggestions of the Japan Gastroenterological Endoscopy Society Guidelines Committee (excluding lesions of the colon). This was conducted to select patients with low risk for LNM to ESD from patients with a high risk of LNM to surgery [21].

For each patient, a histopathological diagnosis was obtained before ESD or surgical treatment. All data from patients who were diagnosed with histology-confirmed gastrointestinal adenocarcinoma or epithelial dysplasia were registered in a database. Patient identifiers were removed to allow analysis without violating sensible patient information.

Ethical approval for this retrospective analysis was obtained from the Regional Research Ethics Committee (AAST GOM Niguarda, Milan). This study was performed in accordance with the Declaration of Helsinki. Due to the use of a de-identified dataset from the database, the institutional review board waived the requirement for individual informed consent.

## 3. EUS Technique

Diagnostic EUS was performed with a linear echoendoscope (Olympus GF-UCT 180, ultrasound frequency 10–7.5 MHz) under conscious sedation by pethidine plus midazolam and saline solution infusion, with assisted ventilation by oxygen at 2 L/min. Each operator was part of a team of gastroenterologists with an overall experience of more than 600 cases of endoscopic ultrasound; as part of the endoscopic team, each member was also mentored by a senior tutor.

**Figure 2:**
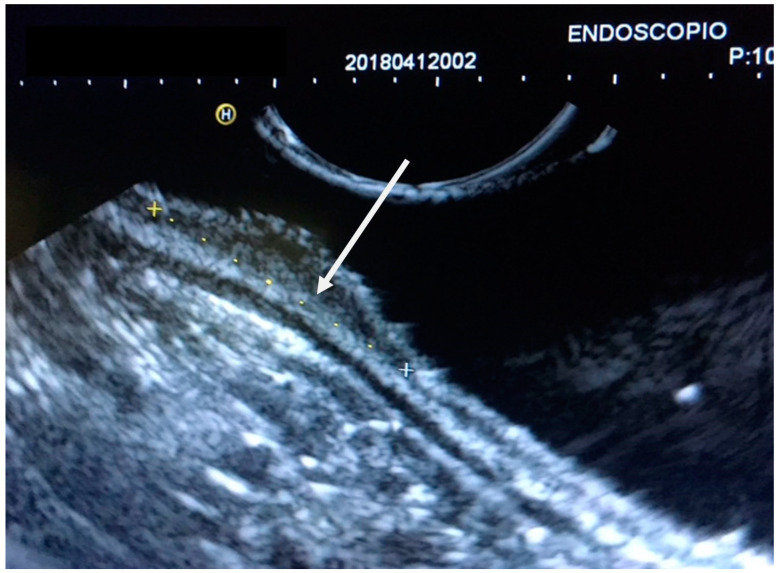
EUS of early gastric cancer at the lesser curvature of the gastric. Antrum with infiltration of the *muscularis mucosae* (arrow). Staging uT1m2, N0.

**Figure 3:**
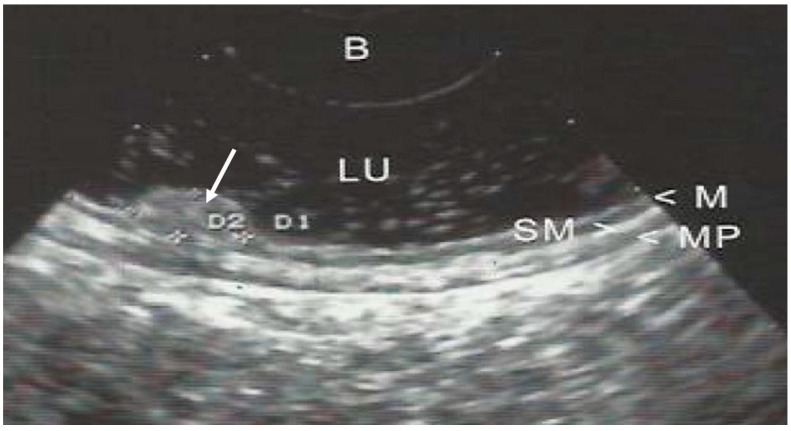
EUS of early gastric cancer (arrow) limited to the lamina propria. Staging uT1m1. (LU—lumen; M—mucosa; SM—submucosa; MP—*muscularis propria*). B—balloon.

**Figure 4:**
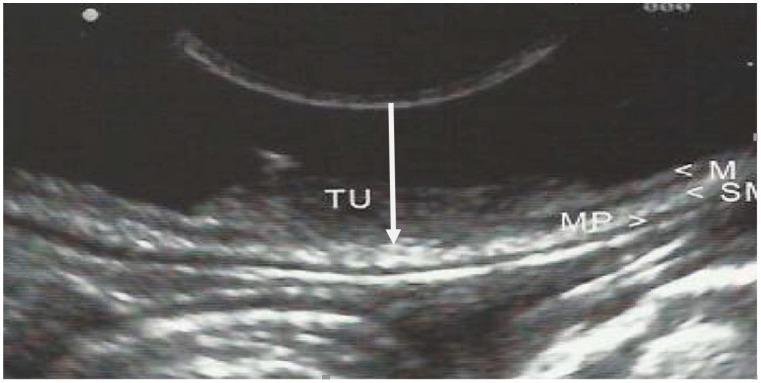
EUS image of early gastric cancer with shallow infiltration (arrow) of the submucosal tissue (staging sm1). (M—mucosa; SM—submucosa; MP—*muscularis propria*; TU—tumor).

The GI wall was divided into 5 layers and examined by EUS; identification of cancer invasion depth by EUS was evaluated as M if the hypoechoic mass disrupted the sonographic layers 1–2, as SM if it disrupted layers 1–3, as *muscularis propria* (MP) if layers 1–4 were disrupted, and as subserosa and serosa (SS) if layers 1–5 were discontinued. (Figure 2, Figure 3 and Figure 4).

## 4. Data Analysis

All the results of the definitive histopathological specimen removed during the endoscopic treatment or after surgical procedures were analyzed and compared with the EUS staging results. The mucosal and submucosal staging was evaluated by preoperative EUS as correctly staged or incorrectly staged (understaged or overstaged).

Accuracy was calculated within the following limitations:

Correctly staged by EUS;

- True-positive lesions with submucosal invasion;
- True negative lesions without submucosal invasion;

Incorrectly staged by EUS;

- False negative lesions with submucosal invasion (understaged by EUS);
- False positive lesions without submucosal invasion (overstaged by EUS).

## 5. Results

### 5.1. Patients Demographics and Tumor Characteristics and Locations

A total of 293 patients were submitted for EUS staging of the GI lesions.

Patients’ demographics, different tumor locations, and EUS layer invasion are shown in Table 2.

The mean age of patients was 63.5 years (22–92 years); 176 patients were male and 117 patients were female. With regard to tumors’ location, 41 cases (13.99%) originated from the esophagus (19 squamous cell carcinomas, 8 adenocarcinomas, and 14 displastic lesions); 106 (36.18%) from the stomach; 121 (41.29%) from the rectum; and 25 cases (8.53%) from other sites. Endoscopic ultrasonographic results were that 155 cases were M lesions (52.90%), which were most prevalent; 32 cases were SM lesions (10.92%); 32 cases were PM lesions (10.92%); and 74 cases were SS lesions (25.25%).

### 5.2. Operative Procedures and Pathological Findings

An ESD procedure was performed on 175 patients (59.72%).

A total of 118 patients underwent surgery, including 21 esophagectomies (7.16%); 10 total gastrectomies (3.41%); 21 subtotal gastrectomies (7.16%); and 66 (22.52%) low anterior resections (Table 3).

Based on the pathological T-stage disease, 136 cases (46.41%) were T1m lesions, 49 cases (16.72%) were T1sm lesions, 34 cases (11.60%) were T2 lesions, and 74 cases (25.25%) were lesions deeper than T2.

### 5.3. Overall Diagnostic Accuracy

EUS for gastrointestinal neoplasia was evaluated for correct and incorrect staging (understaging and overstaging rates).

Overall, EUS accurately staged 242 of 293 lesions (82.59%). EUS understaged 38 of 293 (12.96%) and 25 of these patients received a surgical resection after ESD due to deep infiltration. EUS overstaged 13 of 293 lesions (4.43%) while evaluating for submucosal invasion or deeper.

The accuracy rate of EUS for rectosigmoid and for the stomach was lower with respect to that obtained for duodenum and esophageal lesions, but without statistical significance (*p* = 0.096) (Table 4).

## 6. Discussion

As a result of surgical morbidity, minimally invasive endoscopic therapy such as EMR and ESD has been investigated over the past two decades as treatment options for GI cancers confined to the mucosa or superficial submucosa (SM1).

By examining histologically resected specimens, ESD can determine exactly the depth and free margins of the tumor, the lymphatic and venous invasion, as well as the grade of tumor differentiation.

A biopsy before EUS can cause some distortion of the lesion, but it is generally considered safe. When performed prior to EUS evaluation, it may include the histopathological evaluation, which can be relevant information to confirm the diagnosis before proceeding with EUS.

ESD has a significantly low morbidity rate (1–3%) and mortality rate (0%) and preserves GI function and quality of life [22,23,24,25].

Complete and accurate staging for patients with GI neoplasia provides preoperative differentiation of mucosal and submucosal invasion and is crucial to assessing the extent of the disease. It is therefore possible to recommend a treatment plan that is appropriate for each patient.

Over the past two decades, many studies have investigated the accuracy of EUS in staging superficial esophageal cancer. In staging superficial esophageal cancer, EUS accuracy varies between 33% and 85% [8,9,10].

A total of 12.96% of the GI lesions were understaged by EUS in our study; therefore, patients must be informed that surgical resection may be necessary after ESD in the case of non-curative endoscopic resection.

Aside from its ability to detect neoplastic lesions and determine their stage accurately, EUS has several other advantages.

Since EUS probe is placed close to the lesion, it helps distinguish T1m from T1sm and T1 from T2 lesions. The use of EUS-guided fine-needle aspiration can also enhance EUS’ ability to diagnose locoregional malignant lymph nodes.

EUS can be used before ESD to detect surrounding blood vessels and the degree of fibrosis, helping to predict procedure times and reduce bleeding and perforation risks.

Regarding our priority algorithm using chromoendoscopy, biopsy, and EUS in our study, we are aware that each diagnostic method has its own strengths and limitations.

Chromoendoscopy is a useful tool for identifying mucosal and submucosal abnormalities, but it may not provide enough information to accurately diagnose the depth of invasion of the lesion. A biopsy can provide tissue samples for histological diagnosis, but it may not be accurate in determining the depth of invasion either. EUS, on the other hand, can provide more precise information about the depth of invasion and other characteristics of the lesion, which can be helpful in selecting appropriate patients for endoscopic resection.

There are some endoscopic findings that may negatively influence EUS accuracies, such as microinfiltration of tumors, peri-tumoral surrounding inflammation, severe fibrosis associated with ulcers, benign ulcerous changes, benign cystic changes of the submucosal layer, *muscularis mucosae* deformities, and the location of some lesions such as those of the gastric fundus, recto-sigmoid junction, and anorectal margin [16,17,18]. This could explain why in our study the accuracy rate of EUS staging for recto-sigmoid and stomach lesions was lower in comparison with that obtained for oesophageal neoplasms.

Our study shows that EUS has an overall good accuracy (82.59%) in detecting mucosal or submucosal invasion in GI neoplasms.

A number of factors can affect EUS accuracy, including operator experience and volume, EUS-specific technology, cancer type, and location of the lesion. For suspected superficial GI neoplasms, EUS can change the diagnostic and therapeutic algorithm in the hands of experienced operators. Additionally, we believe that EUS is crucial for detecting T2 lesions that cannot be treated with ESD. Treatment of GI lesions is now individualized and includes either definitive chemoradiation or trimodal therapy, including surgical resection.

At the same time, EUS can diagnose locoregional metastasis including lymph node involvement.

## 7. Conclusions

Our experience in patients with superficial GI neoplasms seems to recommend EUS evaluation, first to rule out infiltration of the *muscularis propria* (T2) and regional lymph node metastases, and secondly to differentiate mucosal from submucosal infiltration.

## Figures and Tables

**Table 1:** Histological distribution of lesions at screening endoscopy.

Variable	N (%)
Noninvasive low-grade neoplasm	81 (27.64%)
Noninvasive high-grade neoplasm	90 (30.71%)
Invasive Adenocarcinoma	114 (38.90%)
Carcinoid tumor	8 (2.73%)

**Table 2:** Clinicopathologic characteristics of 293 patients submitted to EUS (endoscopic ultrasonography) staging for GI lesions.

Variable		N (%)
Gender	Male	176 (60)
	Female	117 (40)
Tumor Location		
Esophagus	Upper third	8 (2.7)
	Middle third	15 (5.1)
	Lower third	18 (2.7)
Stomach	Fundus	12 (4.1)
	Corpus	41 (13.9)
	Antrum	53 (18.1)
Gastro-jejunal anastomosis		4 (1.4)
Duodenum		13 (4.4)
Sigmoid colon		8 (6.1)
Rectum		121 (41.3)
EUS layer invasion	Mucosa	155 (52.9)
	Submucosa	32 (10.9)
	Proper muscle	32 (10.9)
	Subserosa	74 (25.3)

**Table 3:** Operative procedure and pathological findings (N = 293).

Variable		N (%)
Operative procedure	ESD (endoscopic submucosal dissection)	175 (59.7)
	Esophagectomy	21 (7.2)
	Total gastrectomy	10 (3.4)
	Distal subtotal gastrectomy	21 (7.2)
	Lower anterior resection	66 (22.5)
Histological tumor depth	Mucosa	136 (46.4)
	Submucosa	49 (16.7)
	Proper muscle	34 (11.6)
	Subserosa	64 (21.8)
	Serosa exposure	10 (3.4)

**Table 4:** The proportion of corrected diagnosis for tumor depth by EUS compared with the pathologic results. Values are reported as numbers (%).

Tumor Location	N	Accuracy Correctly Diagnosed	Incorrectly Diagnosed	Understaged	Overstaged
Esophagus	41	39 (95.1)	2 (4.9)	2 (4.9)	-
Stomach	106	88 (83)	18 (16.9)	15 (14.1)	3 (2.8)
Duodenum	13	13 (100)	-	-	-
Gastro-jejunal anastomosis	4	3 (75)	1 (25)	1 (25)	-
Sigmoid colon	8	6 (75)	2 (25)	2 (25)	-
Rectum	121	93 (76.9)	28 (23.1)	18 (14.9)	10 (8.3)
Total	293	242 (82.6)	51 (17.4)	38 (12.9)	13 (4.4)

## Data Availability

The data are not publicly available because of privacy and ethical restrictions. The data presented in this study are available upon request from the first author, P.G.

## Abbreviations

EUS—endoscopic ultrasound; GI—gastrointestinal; ESD—endoscopic submucosal dissection; EMR—endoscopic mucosal resection; CLS—colonoscopy; LMN—lymph node metastasis; SM—submucosa; M—mucosa; PM—proper muscle; SS—subserosa; EGC—early gastric cancer; EGD—esophagogastroduodenscopy; SEC—superficial esophageal cancer; LU—lumen; MP—*muscolaris propria*; TU—tumor.
